# Food groups and nutrients consumption and risk of endometriosis: a systematic review and meta-analysis of observational studies

**DOI:** 10.1186/s12937-022-00812-x

**Published:** 2022-09-22

**Authors:** Arman Arab, Elham Karimi, Kristina Vingrys, Mahnaz Rezaei Kelishadi, Sanaz Mehrabani, Gholamreza Askari

**Affiliations:** 1 Department of Community Nutrition, School of Nutrition and Food Science, Food Security Research Center, Isfahan University of Medical Sciences, Isfahan, Iran; 2 Department of Clinical Nutrition, School of Nutrition and Food Science, Food Security Research Center, Isfahan University of Medical Sciences, Isfahan, Iran; 3 Research Development Center, Arash Women’s Hospital, Tehran University of Medical Sciences, Tehran, Iran; 4 Institute for Health and Sport, Victoria University, PO Box 14428, Melbourne, Victoria 8001 Australia

**Keywords:** Food groups, Diet, Endometriosis, Systematic review, Meta-analysis

## Abstract

Dietary factors may play a role in the etiology of endometriosis and dietary intake of some food groups and nutrients could be associated with endometriosis risk. This systematic review and meta-analysis of observational studies was conducted to summarize the findings on the association between dietary intakes of selected food groups and nutrients (dairy, fats, fruits, vegetables, legumes, and animal-derived protein sources), and the risk of endometriosis among adult women. PubMed, Scopus, and ISI Web of Science were systematically searched up to September 2022. The inverse variance-weighted fixed-effect method was used to estimate the effect size and corresponding 95% CI. A total of 8 publications (4 studies) including 5 cohorts and 3 case-control with a sample size ranging from 156 to 116,607 were included in this study. A higher intake of total dairy [all low-fat and high-fat dairy foods] was associated with decreased risk of endometriosis (RR 0.90; 95% CI, 0.85 to 0.95; *P* < 0.001; I^2^ = 37.0%), but these associations were not observed with intakes of low or high-fat dairy, cheese or milk. Increased risk of endometriosis was associated with higher consumption of red meat (RR 1.17; 95% CI, 1.08 to 1.26; *P* < 0.001; I^2^ = 82.4%), trans fatty acids (TFA) (RR 1.12; 95% CI, 1.02 to 1.23; *P* = 0.019; I^2^ = 73.0%), and saturated fatty acids (SFA) (RR 1.06; 95% CI, 1.04 to 1.09; *P* < 0.001; I^2^ = 57.3%). The results of this meta-analysis suggest that there may be differing associations between dietary intake of dairy foods, red meat, SFAs, and TFAs and the risk of endometriosis. It may be useful to extend the analysis to other types of food groups and dietary patterns to obtain a complete picture. Additionally, further investigations are needed to clarify the role of diet in the incidence and progression of endometriosis.

**Trial registration:** PROSPERO, CRD42020203939.

## Introduction

Endometriosis is a gynecological condition defined as the presence of endometrial glands and stroma-like lesions outside the uterus that can cause infertility and severe debilitating pain [1]. The prevalence of endometriosis in women of reproductive age is approximately 10 to 15, and 70% of women with pelvic pain are reported to suffer from endometriosis [1]. Endometriosis is an estrogen-dependent disorder associated with symptoms such as severe menstrual bleeding and pain, pelvic pain, and subfertility [2]. The etiology of endometriosis is multifaceted and not fully understood, however, genetic, anatomic, immunologic, hormonal, and environmental factors (e.g. exercise and diet) can play a substantial role in the pathogenesis of this condition [3].

Dietary factors can be related to endometriosis etiology due to their role in regulating steroid hormone metabolism, the effect on muscle contraction, regulating inflammation, oxidative stress, and the menstrual cycle [4]. For instance, some observational studies showed that a plant-based diet leads to a decrease in the bioavailability of estrogen, estrogen plays a role in inducing extra-uterine endometrial tissue proliferation [5, 6]. Also, higher consumption of fruits and green vegetables may be protective because it can decrease inflammatory markers such as interleukin-6 (IL-6) that are elevated among women diagnosed with endometriosis [7]. Dairy products contain anti-inflammatory and anti-oxidative agents, vitamin D, and calcium that may induce a protective effect in endometriosis [8]. Also, the composition of fatty acids in the diet may be related to the risk of endometriosis [4]. In this regard, fish oil consumption may lead to a decreased risk of endometriosis due to its anti-inflammatory effects, while high trans-fat intake leads to an increased risk of endometriosis [2, 4]. Red meat is another dietary factor that may be related to an increased risk of endometriosis because it can affect estrogen levels [9] that are involved in the pathogenesis of endometriosis by promoting the expression and release of pro-inflammatory factors [10].

Dietary factors may be associated with the progression and development of endometriosis, but the findings are contradictory due to various issues including different study designs, sample size, and other methodological aspects. For instance, some observational studies did not show any significant association between dairy intake and endometriosis risk [2, 7] while others demonstrated that dairy intake is a protective factor [11]. Similarly, whilst some observational studies did not demonstrate any association between endometriosis risk and consumption of red meat and fish [2], other studies showed that a higher intake of red meat and a lower intake of fish were related to an elevated risk of endometriosis [7, 12].

Although a previous literature review examined the relationship between diet and endometriosis risk, that study searched the literature up to 2011 and also overlooked a meta-analysis on this topic. Therefore, conducting a more comprehensive systematic review and meta-analysis of this issue was necessary. Therefore, the current study was conducted to summarize the findings on the association between dietary food groups and nutrient consumption and the risk of endometriosis using a systematic review and meta-analysis of observational studies among adult women.

## Methods

### Data source and search strategy

The present study was designed and conducted based on the Preferred Reporting Items for Systematic Reviews and Meta-Analysis (PRISMA) Statements [13] and also was registered (Prospero database: CRD42020203939). The bibliographic databases PubMed, Scopus, and ISI Web of Science were searched from the earliest available date to September 2022 to identify relevant studies. Two reviewers (A.A and E.K) independently searched the databases to identify studies investigating the association between dietary intake of selected food groups and nutrients and risk of endometriosis, using the following keywords: (endometriosis OR endometrioses OR endometriomas OR endometrioma) AND (diet OR “diet type” OR “dietary habit” OR “dietary pattern” OR “eating pattern” OR foods OR nutrition OR “diet quality” OR “food groups”) (Table 1). The reference lists of the final articles were also checked to identify any additional eligible studies that had not been captured via the database searches.

**Table 1:** Search terms

**PubMed**	
Search hits: 648 ((“endometriosis”[MeSH Terms] OR “endometriosis”[All Fields]) OR “endometrioses”[All Fields]) AND ((((((((((((“diet”[MeSH Terms] OR “diet”[All Fields]) OR “diet type”[All Fields]) OR “dietary habit”[All Fields]) OR “dietary pattern”[All Fields]) OR “eating pattern”[All Fields]) OR (((“food”[MeSH Terms] OR “food”[All Fields]) OR “foods”[All Fields]) OR “food s”[All Fields])) OR (((((((((((“nutrition s”[All Fields] OR “nutritional status”[MeSH Terms]) OR (“nutritional”[All Fields] AND “status”[All Fields])) OR “nutritional status”[All Fields]) OR “nutrition”[All Fields]) OR “nutritional sciences”[MeSH Terms]) OR (“nutritional”[All Fields] AND “sciences”[All Fields])) OR “nutritional sciences”[All Fields]) OR “nutritional”[All Fields]) OR “nutritionals”[All Fields]) OR “nutritions”[All Fields]) OR “nutritive”[All Fields])) OR “diet quality”[All Fields]) OR “food groups”[All Fields]))	
**Scopus**	
Search hits: 940 ((TITLE-ABS-KEY (endometriosis) OR TITLE-ABS-KEY (endometrioses) OR TITLE-ABS-KEY (endometriomas) OR TITLE-ABS-KEY (endometrioma))) AND ((TITLE-ABS-KEY (diet) OR TITLE-ABS-KEY (“diet type”) OR TITLE-ABS-KEY (“dietary habit”) OR TITLE-ABS-KEY (“dietary pattern”) OR TITLE-ABS-KEY (“eating pattern”) OR TITLE-ABS-KEY (food) OR TITLE-ABS-KEY (foods) OR TITLE-ABS-KEY (nutrition) OR TITLE-ABS-KEY (“diet quality”) OR TITLE-ABS-KEY (“food groups”)))	
**Web of Science**	
Search hits: 423 (TS = (endometriosis) OR TS = (endometrioses) OR TS = (endometriomas) OR TS = (endometrioma)) AND (TS = (diet) OR TS = (“diet type”) OR TS = (“dietary habit”) OR TS = (“dietary pattern”) OR TS = (“eating pattern”) OR TS = (foods) OR TS = (nutrition) OR TS = (“diet quality”) OR TS = (“food groups”))	

### Study selection and eligibility criteria

The PI(E) COS model was used to determine eligibility criteria, representing Population (aged > 18 years old women), Exposure (high dietary intake of selected food groups and nutrients [i.e., the last tertile, quartile, or quintile of the dietary intakes]), Comparison (low dietary intake of selected food groups and nutrients [i.e., the first tertile, quartile, or quintile of the dietary intake]), Outcome (risk of endometriosis), and Study design (case-control or cohort studies).

To be included in our study, articles investigating the association between dietary food groups and nutrients and the risk of endometriosis had to meet the following criteria: (1) original human observational studies either with case-control or cohort design; (2) published in the English language; (3) which reported at least one of the intended food groups or nutrients including fat, red meat, poultry, fish, total fruits, total vegetables, total dairy, milk, cheese, egg, and legumes as exposure in association with endometriosis risk as an outcome.

The exclusion criteria were as follows: (1) pre-clinical studies; (2) females aged < 18 years old; and (3) not original full-length articles including poster abstracts, case reports, review articles, editorials, or without original data or articles with no appropriate outcome measures. Two assessors independently (A.A and E.K) conducted the selection process. Any disagreement was resolved through discussion with a third reviewer (G.A).

### Data extraction

The following data were extracted: the first author’s name, published year, study location, sample size, participant characteristics [including age and body mass index (BMI)], study design, endometriosis diagnosis method, type of dietary assessment approach, and statistical adjustment.

### Quality assessment

The quality assessment of eligible studies was performed by two reviewers (A.A and E.K) individually using the Newcastle-Ottawa Scale (NOS) star system (ranged, 0–9 stars) [14], which focuses on selection, comparability, and outcome. Studies scoring ≥7, 4–6, and ≤ 3 points were assumed as high, moderate, and low quality, respectively [15].

### Statistical analysis

Relative risks (RR) or odds ratio (OR) was used to measure the relationship between dietary consumption of each food group or nutrient and endometriosis risk. OR or RR in every study was converted to effect size by using their natural logarithms, and the standard errors (SEs) were calculated from these logarithmic numbers and their corresponding 95% confidence interval (CI). Since the outcome (endometriosis) occurs relatively infrequently (< 20%), the meta-analysis was based on the assumption that all measures are RRs [16, 17]. The inverse variance-weighted fixed-effect method was used to estimate the effect size and corresponding 95% CI [18]. Heterogeneity between effect size of included studies was estimated by the chi-squared (χ^2^) test and I^2^ statistic [*I* ^2^ index < 40 (low heterogeneity), 40–75 (moderate heterogeneity) and > 75% (high heterogeneity)] [19]. The low number of included studies in each analysis precludes us to conduct sub-group analysis. Sensitivity analyses were performed to assess each study’s influence on the stability of the meta-analysis results. Each time, one study was excluded to show that study’s impact on the combined effect estimate. Publication bias was assessed using Egger’s and Begg’s statistics [20]. When publication bias was found, trim and fill analysis was performed to adjust for potential publication bias on overall effect size. A *P*-value < 0.05 was considered statistically significant. The statistical analyses were done using STATA statistical program version 11.2 (Stata Corporation, College Station, TX, USA).

## Results

### Characteristics of included studies

**Fig. 1:**
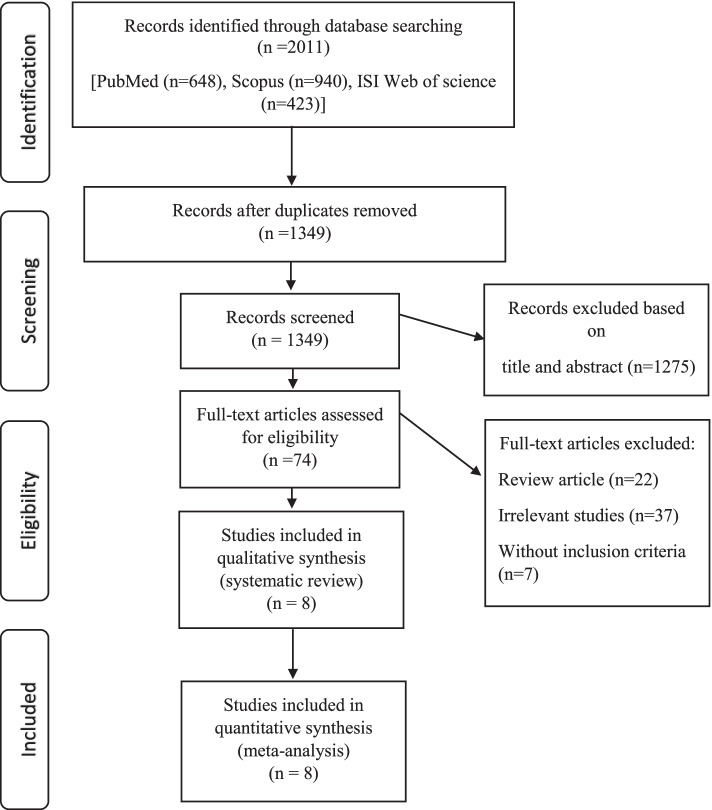
The flow diagram of study selection

A total of 8 publications (4 studies) were included in this systematic review and meta-analysis with a sample size ranging from 156 to 116,607. Participants’ mean age at the baseline of studies ranged from 18 to 41.38 years. The included studies were conducted between 2004 and 2020. Among included publications, six were from the United States [2, 4, 8, 11, 12, 21], one from Italy [7], and one from Iran [22]. Four publications [4, 8, 11, 12] reported the baseline mean BMI of participants and the others [2, 7, 21, 22] only report the number of participants across different categories of BMI. Moreover, five publications [4, 8, 11, 12, 21] were cohort in design, and three were case-control [2, 7, 22]. All of the included publications [2, 4, 7, 8, 11, 12, 21, 22] utilized a laparoscopic approach to identify endometriosis. In all publications, the dietary intakes were examined by a food frequency questionnaire (ranging from 122 to 147 food items). Four publications examined the dietary intakes of participants after enrollment through multiple timepoints (1991, 1995, 1999, 2003) [4, 11, 12, 21], three studies examined this issue upon participants enrollment [2, 7, 23], and Nodler et al. [8] asked retrospectively about diet during adolescence. Moreover, all of the publications adjusted total energy intake except the work of Parazzini et al. [7]. Based on the NOS, all of the enrolled studies were ranked as high quality. The works of Missmer et al. [4], Harris et al. [11, 21], Yamamoto et al. [12], and Nodler et al. [8] were published based on the data of the Nurses’ Health Study II cohort. The study selection process and the details of the final studies are summarized in Fig. 1 and Table 2, respectively.

**Table 2:** Characteristics of included studies

Author, Year	Location	Sample size	Age (Mean)	BMI (kg/m2)	Study Design	Type of diagnosis	Type of dietary assessment	Statistical adjustments	Reported exposure	Quality assessment
Missmer et al., 2010 [4]	US	116,607	35.64	25≤	Cohort	Laparoscopy	FFQ/130 Items	age at menarche, length of menstrual cycle, parity, BMI, energy intake	Total fat, MUFA, PUFA, SFA, TFA	High
Trabert et al., 2011 [2]	US	944	18–49	NM	Case–control	Laparoscopy	FFQ/122 items	Age and year of enrolment, energy intake, income, BMI, smoking and alcohol consumption	Dairy, total fat, MUFA, PUFA, SFA, TFA, fruits, vegetables, fish, poultry, red meat	High
Harris et al., 2013 [11]	US	70,556	35.91	25≤	Cohort	Laparoscopy	FFQ/130 Items	age, age at menarche, length of menstrual cycle, parity, BMI, energy intake	Dairy, high-fat dairy, low-fat dairy, cheese, milk	High
Yamamoto et al., 2018 [12]	US	116,429	36.23	25≤	Cohort	Laparoscopy	FFQ/130 Items	race, age at menarche, length of menstrual cycle between ages 18–22, parity, body mass index, energy intake, recent gynecologic exam	Egg, fish, poultry, red meat	High
Harris et al., 2018 [21]	US	70,835	36.08	NM	Cohort	Laparoscopy	FFQ/130 Items	age, age at menarche, length of menstrual cycle, parity, BMI, energy intake	Fruits, vegetables	High
Nodler et al., 2020 [8]	US	116,429	41.38	25.68	Cohort	Laparoscopy	FFQ/124 items	body mass index at age 18 years, age at menarche, adolescent physical activity, smoking in adolescence, adolescent hormonal contraceptive use, and energy intake.	Dairy, high-fat dairy, low-fat dairy, cheese, milk	High
Parazzini et al., 2004 [7]	Italy	1008	33	NM	Case-control	Laparoscopy	FFQ	age, calendar year at interview, education, parity, body mass index.	Cheese, milk, fruits, vegetables, egg, fish, red meat	High
Youseflu et al., 2020 [23]	Iran	156	30.18	NM	Case-control	Laparoscopy	FFQ/147 Items	Age, energy intake, BMI, income	Dairy, high-fat dairy, low-fat dairy, total fat, MUFA, PUFA, SFA, TFA, fruits, vegetables, egg, fish, poultry, red meat	High

*US* United States, *NM* Not mentioned, *FFQ* Food Frequency Questionnaire, *BMI* Body Mass Index, *MUFA* Monounsaturated Fatty Acids, *PUFA* Polyunsaturated Fatty Acids, *SFA* Saturated Fatty Acids, *TFA* Trans Fatty Acid

### Finding from meta-analysis

#### The association between dietary intake of dairy foods (total-, low-, and high-fat dairy, cheese, and milk) and risk of endometriosis

**Fig. 2:**
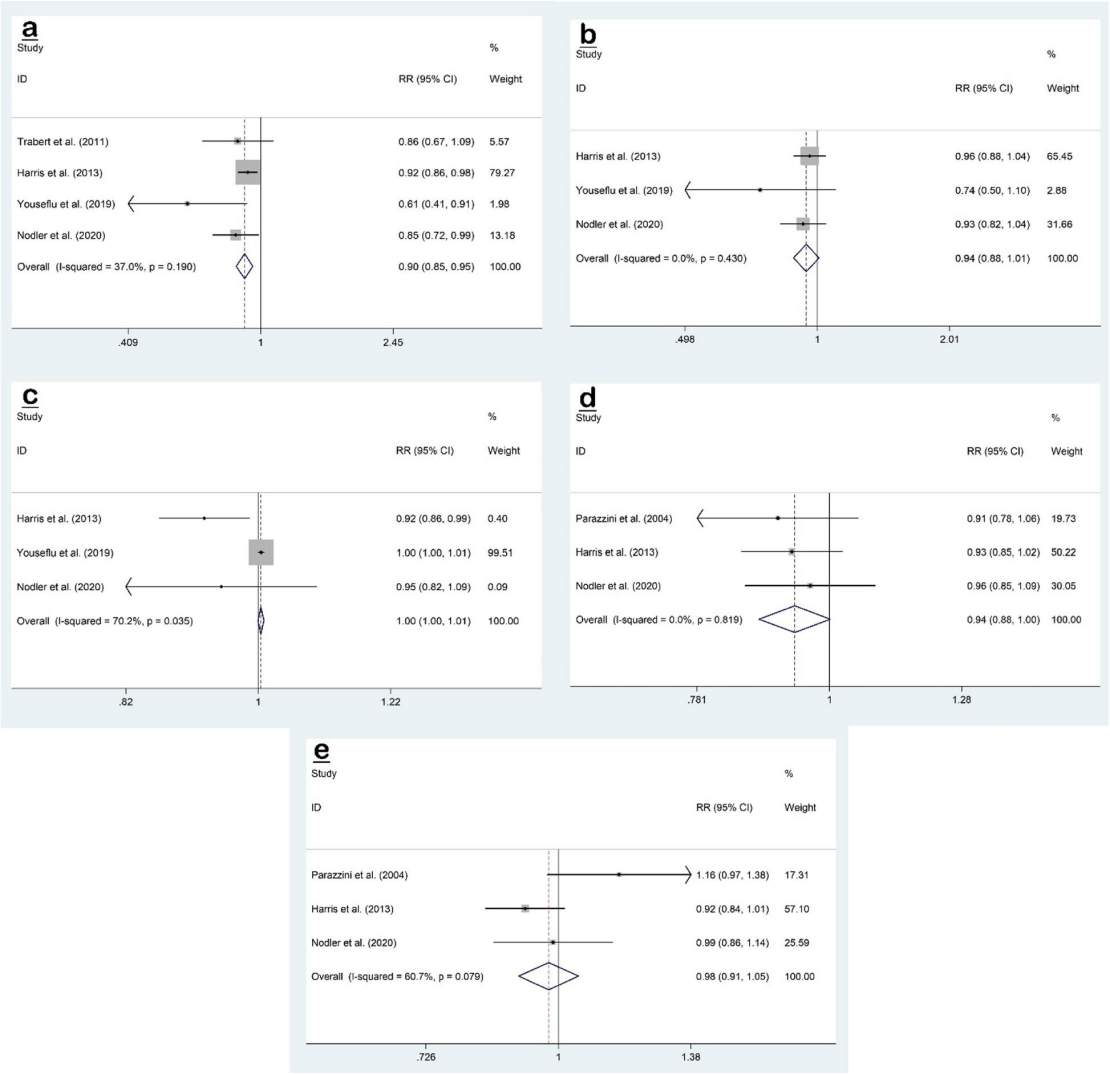
Forest plots of the association between dietary intake of dairy foods (total-(**a**), low-(**b**) and high-fat(**c**) dairy, cheese(**d**) and milk(**e**)) and risk of endometriosis

The pooled effect size of four datasets [2, 8, 11, 22] of the association between total dairy (all low-fat and high-fat dairy foods) intake and endometriosis risk was RR 0.90; 95% CI, 0.85 to 0.95; *P* < 0.001, with no evidence of significant heterogeneity (I^2^ = 37.0%, *P* = 0.190) (Fig. 2a).

Three studies [8, 11, 22] evaluated the association between high-fat dairy (whole milk, cream, ice cream, cream cheese, other cheese, and butter) intake and risk of endometriosis, but the pooled effect size showed no association (RR 0.94; 95% CI, 0.88 to 1.01; *P* = 0.083), and was without between-study heterogeneity (I^2^ = 0.0%, *P* = 0.430) (Fig. 2b).

Three studies [8, 11, 22] assessed the relationship between low-fat dairy (skim/low-fat milk, sherbet, yogurt, and cottage cheese) intake and risk of endometriosis, and no association was observed (RR 1.00; 95% CI, 1.00 to 1.01; *P* = 0.073), with evidence of significant heterogeneity (I^2^ = 70.2%, *P* = 0.035) (Fig. 2c).

Three studies [7, 8, 11] investigated the association between cheese intake and risk of endometriosis, and no association was observed (RR 0.94; 95% CI, 0.88 to 1.00; *P* = 0.055), with no evidence of heterogeneity (I^2^ = 0.0%, *P* = 0.819) (Fig. 2d).

The association between milk intake and risk of endometriosis was evaluated in three studies [7, 8, 11] and the pooled effect size showed no association (RR 0.98; 95% CI, 0.91 to 1.05; *P* = 0.509) with significant heterogeneity (I^2^ = 60.7%, *P* = 0.079) (Fig. 2e).

No evidence of publication bias was observed for total-dairy (Begg’s test: *P* = 0.174, Egger’s test: *P* = 0.087), low-fat dairy (Begg’s test: *P* = 0.602, Egger’s test: *P* = 0.308), cheese (Begg’s test: P = 0.602, Egger’s test: *P* = 0.855) and milk (Begg’s test: *P* = 0.117, Egger’s test: *P* = 0.217). As there was evidence of publication bias for high-fat dairy (Begg’s test: P = 0.117, Egger’s test: *P* = 0.029), we conducted trim and fill analysis to determine any potentially missed studies, however, no study was added.

The results of the sensitivity analysis for total dairy, high-fat dairy, cheese, and milk showed that the omission of each study did not affect the overall outcome and that the overall findings were not influenced by a particular study. On the other hand, the meta-analysis result for low-fat dairy was sensitive to Youseflu et al. [22] (RR 0.85; 95% CI, 0.74 to 0.97) study.

#### The association between dietary intake of fat (total fat, MUFA, PUFA, SFA, and TFA) and risk of endometriosis

**Fig. 3:**
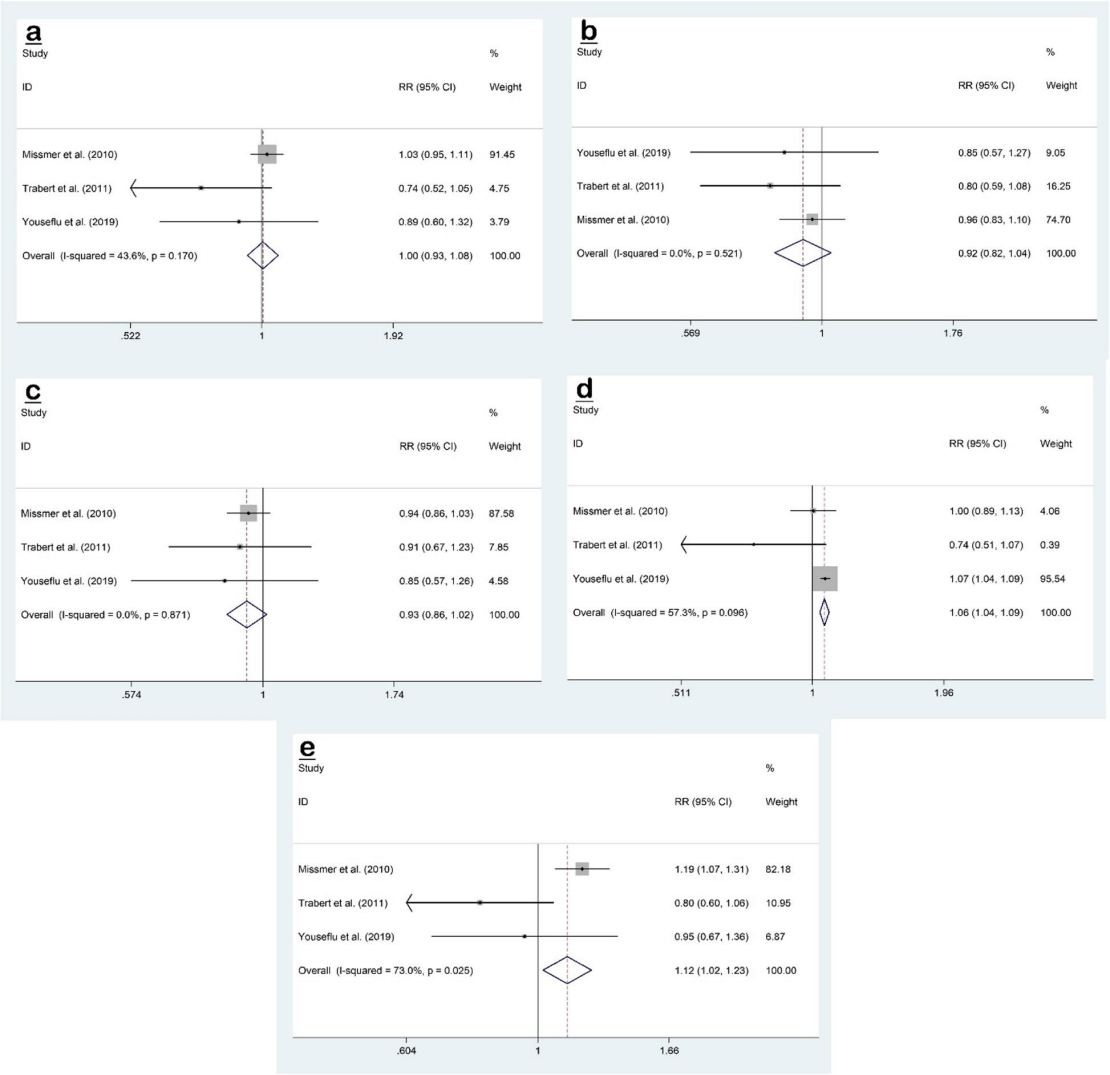
Forest plots of the association between dietary intake of fats (total fat (**a**), MUFA (**b**), PUFA (**c**), SFA (**d**), and TFA (**e**)) and risk of endometriosis

Three studies [2, 4, 22] reported the association between total fat intake and risk of endometriosis, for which our meta-analysis showed no association (RR 1.00; 95% CI, 0.93 to 1.08; *P* = 0.907) with no evidence of significant heterogeneity (I^2^ = 43.6%, *P* = 0.170) (Fig. 3a).

Similarly, three studies [2, 4, 22] that reported dietary intake of monounsaturated fatty acid (MUFA) showed no association (RR 0.92; 95% CI, 0.82 to 1.04; *P* = 0.190) without evidence of heterogeneity (I^2^ = 0.0%, *P* = 0.521) (Fig. 3b).

The pooled effect size of three studies [2, 4, 22] reported no association between intake of polyunsaturated fatty acid (PUFA) and endometriosis risk (RR 0.93; 95% CI, 0.86 to 1.02; *P* = 0.114) with no evidence of significant heterogeneity (I^2^ = 0.0%, *P* = 0.871) (Fig. 3c).

Three studies [2, 4, 22] evaluated the association between saturated fatty acid (SFA) intake and risk of endometriosis, for which our meta-analysis showed a significant relationship (RR 1.06; 95% CI, 1.04 to 1.09; *P* < 0.001), with evidence of significant heterogeneity (I^2^ = 57.3%, *P* = 0.096) (Fig. 3d).

The overall result of a meta-analysis of three studies [2, 4, 22] investigating the association between trans fatty acid (TFA) intake and risk of endometriosis showed a significant association (RR 1.12; 95% CI, 1.02 to 1.23; *P* = 0.019), with significant heterogeneity (I^2^ = 73.0%, *P* = 0.025) (Fig. 3e).

No evidence of publication bias was observed for total fat (Begg’s test: *P* = 0.602, Egger’s test: *P* = 0.290), MUFA (Begg’s test: P = 0.602, Egger’s test: *P* = 0.311), PUFA (Begg’s test: *P* = 0.117, Egger’s test: *P* = 0.198), SFA (Begg’s test: P = 0.117, Egger’s test: *P* = 0.139) or TFA (Begg’s test: P = 0.602, Egger’s test: *P* = 0.295).

The results of the sensitivity analysis for total fat, MUFA, and PUFA showed that the omission of each study did not affect the overall outcome and that the overall findings were not influenced by a particular study. On the other hand, the meta-analysis result for SFA was sensitive to Youseflu et al. [22] (RR 0.95; 95% CI, 0.74 to 1.20) and TFA to Missmer et al. [4] (RR 0.70; 95% CI, 0.42 to 1.14) study.

#### The association between dietary intake of total fruits and total vegetables and the risk of endometriosis

**Fig. 4:**
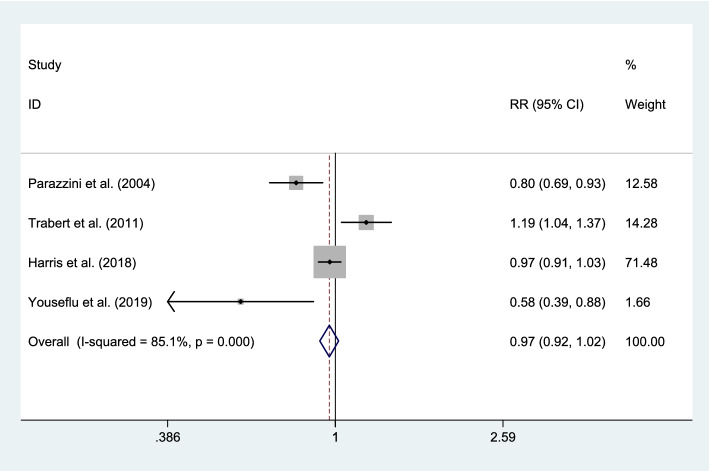
Forest plot of the association between dietary intake of fruits and risk of endometriosis

Quantitative analysis of total fruits intake in four databases [2, 7, 21, 22] showed no association with endometriosis risk (RR 0.97; 95% CI, 0.92 to 1.02; *P* = 0.209). Also, there was evidence of significant heterogeneity between the effect sizes of included studies (I^2^ = 85.1%, *P* < 0.001). Overall, the meta-analysis result was sensitive to Trabert et al. [2] (RR 0.87; 95% CI, 0.75 to 0.97) study (Fig. 4).

**Fig. 5:**
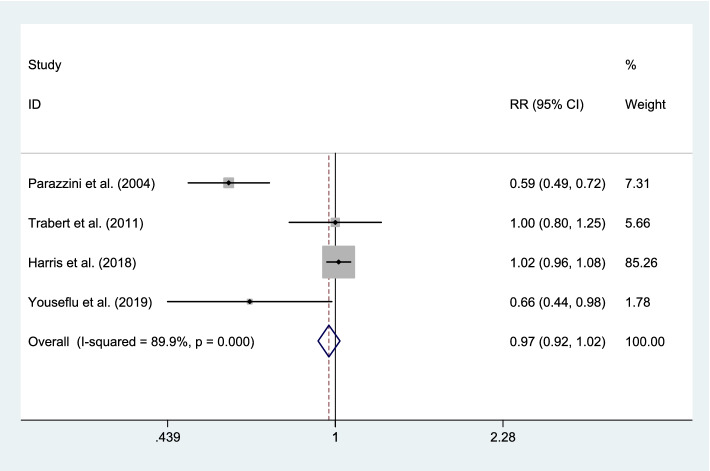
Forest plot of the association between dietary intake of vegetables and risk of endometriosis

Similarly, four studies [2, 7, 21, 22] that reported total vegetables intake showed no association (RR 0.97; 95% CI, 0.92 to 1.02; *P* = 0.256), with evidence of significant heterogeneity (I^2^ = 89.9%, P < 0.001). Overall, the meta-analysis result was sensitive to Harris et al. [21] (RR 0.50; 95% CI, 0.36 to 0.69) study (Fig. 5).

No evidence of publication bias was observed for fruits (Begg’s test: *P* = 0.497, Egger’s test: *P* = 0.610) and vegetables (Begg’s test: P = 0.497, Egger’s test: *P* = 0.287).

#### The association between dietary intake of legumes and risk of endometriosis

**Fig. 6:**
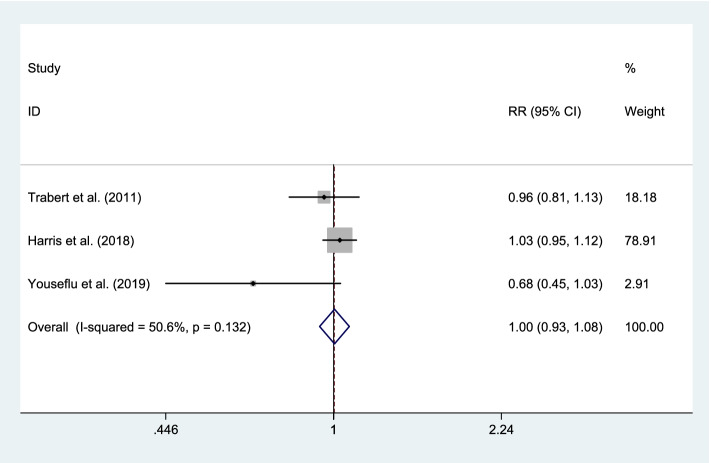
Forest plot of the association between dietary intake of legumes and risk of endometriosis

The relationship between dietary intake of legumes and risk of endometriosis was assessed in three studies [2, 21, 22], for which our meta-analysis did not show a significant association (RR 1.00; 95% CI, 0.93 to 1.08; *P* = 0.921), with no evidence of significant heterogeneity (I^2^ = 50.6%, *P* = 0.132). Overall, the meta-analysis result was not sensitive to individual studies. No evidence of publication bias was also observed (Begg’s test: *P* = 0.117, Egger’s test: *P* = 0.096) (Fig. 6).

#### The association between dietary intake of animal-derived protein sources (red meat, fish, poultry, and egg) and the risk of endometriosis

**Fig. 7:**
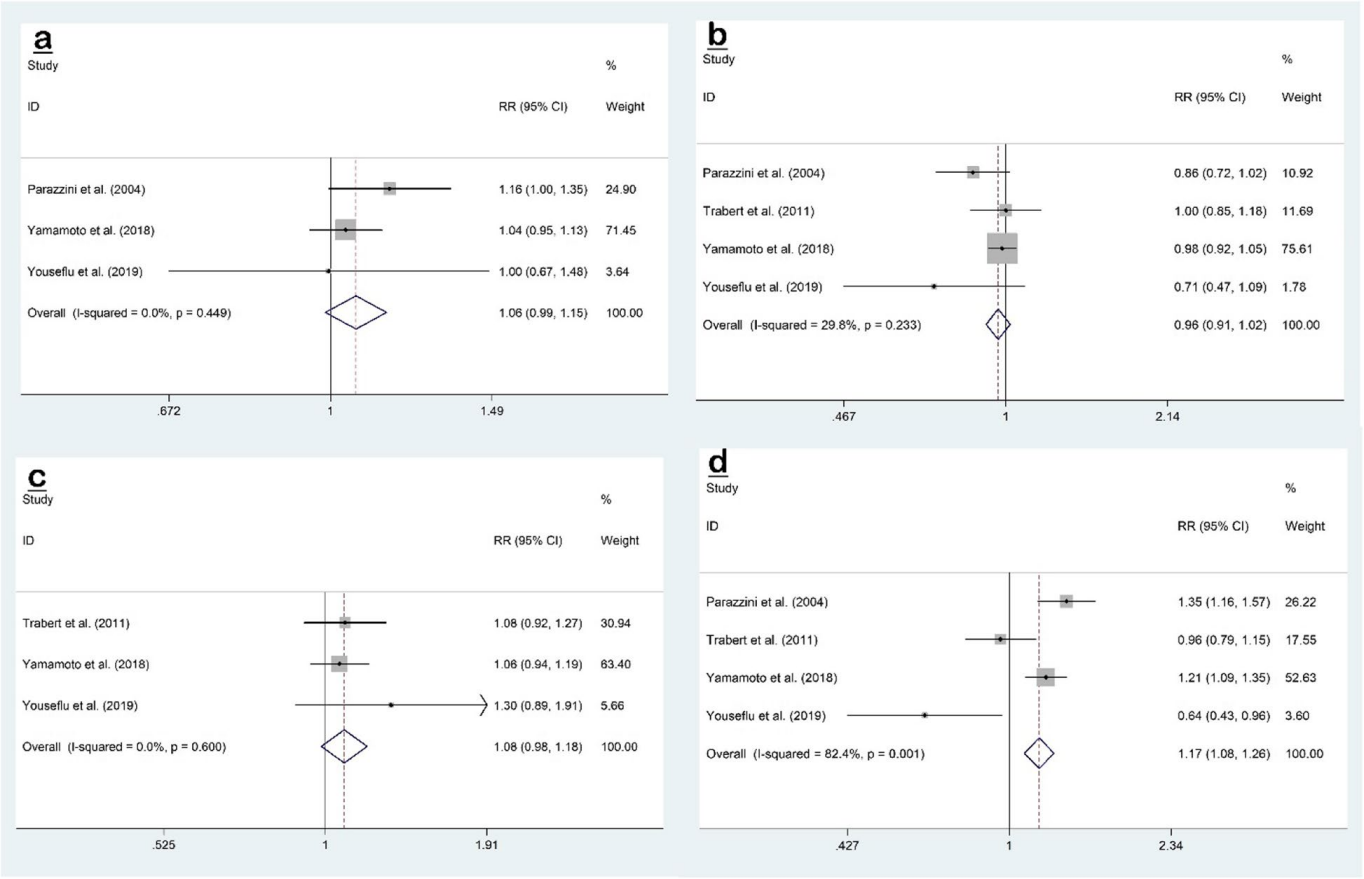
Forest plots of the association between dietary intake of animal-derived protein sources (egg (**a**), fish (**b**), poultry (**c**), and red meat (**d**)) and risk of endometriosis

The pooled effect size of three datasets [7, 12, 22] of the association between egg consumption and risk of endometriosis was RR 1.06; 95% CI, 0.99 to 1.15; *P* = 0.10. Also, there was no evidence of heterogeneity between the effect sizes of included studies (I^2^ = 0.0%, *P* = 0.449) (Fig. 7a).

Four studies [2, 7, 12, 22] investigated the association between fish consumption and endometriosis risk, but the pooled effect size showed no relationship (RR 0.96; 95% CI, 0.91 to 1.02; *P* = 0.208), with no evidence of significant heterogeneity (I^2^ = 29.8%, *P* = 0.233) (Fig. 7b).

Three studies [2, 12, 22] examined the association between poultry intake and risk of endometriosis, and no relationship was observed (RR 1.08; 95% CI, 0.98 to 1.18; *P* = 0.104). Also, there was no evidence of heterogeneity between the effect sizes of included studies (I^2^ = 0.0%, *P* = 0.60) (Fig. 7c).

The pooled effect size of four studies [2, 7, 12, 22] indicated a significant association between red meat intake and risk of endometriosis (RR 1.17; 95% CI, 1.08 to 1.26; *P* < 0.001), with evidence of significant heterogeneity (I^2^ = 82.4%, *P* = 0.001) (Fig. 7d).

No evidence of publication bias was observed for studies reporting dietary intake of egg (Begg’s test: *P* = 0.602, Egger’s test: *P* = 0.884), fish (Begg’s test: *P* = 0.174, Egger’s test: *P* = 0.222), poultry (Begg’s test: *P* = 0.117, Egger’s test: *P* = 0.096), and red meat (Begg’s test: P = 0.174, Egger’s test: *P* = 0.214).

The results of the sensitivity analysis for fish, poultry, and meat showed that the omission of each study did not affect the overall outcome and that the overall findings were not influenced by a particular study. On the other hand, the meta-analysis result for the egg was sensitive to Trabert et al. [2] (RR 0.87; 95% CI, 0.75 to 0.97) study.

## Discussion

This systematic review and meta-analysis was conducted to summarize the findings on the association between dietary intakes of selected food groups and nutrients and the risk of endometriosis. Our findings suggest that a high intake of total dairy may be associated with decreased risk of endometriosis. Furthermore, high consumption of red meat, SFA, and TFA was associated with an increased risk of endometriosis. These findings suggest that dietary factors may play a role in the risk of endometriosis.

The findings on the relationship between total dairy intake and the risk of endometriosis were in agreement with previous reports [8, 23], however, differences in dietary intake assessment of Parazzini et al. [7] may be a possible cause of controversy between the findings. Dietary data reported in Trabert et al. [2], and Youseflu et al. [22] studies were assessed by an FFQ to determine the participants’ intakes during the previous year; however, Nodler et al. [8] used FFQ to examine their intakes during adolescence. Whereas the study by Parazzini et al. [7] assessed the dietary intake of participants by interview, where women were asked to report the number of portions/week of selected food items in the year before the interview. Potential mechanisms associated with the inverse relationship between dairy intake and risk of endometriosis may be related to calcium and vitamin D content of dairy foods and their potential role in the down-regulation of growth-promoting factors, such as insulin-like growth factor-I and up-regulation of negative growth factor modulators, such as transforming growth factor β [2]. It has also been shown that inflammatory factors such as reactive oxygen species (ROS), tumor necrosis factor-α (TNF-α), and IL-6 were all reduced by a higher calcium and dairy intake [24]. The anti-inflammatory effects of vitamin D have also been shown by reducing C-reactive protein (CRP) [25]. Furthermore, casein and whey protein in milk products is associated with anti-inflammatory, anti-carcinogenic, and immunomodulation activity [26]. A high-protein diet may be involved in reducing the risk of endometriosis by modulating endocrine and immune system functions, as well as weight loss [26].

Our analysis also showed no association between total fat, MUFA, and PUFA intake and an increased risk of endometriosis associated with dietary intake of SFA and TFA. Missmer et al. [4] in a prospective study reported that total fat intake was not associated with endometriosis, and TFA is associated with an increased risk of endometriosis; however, intake of long-chain omega-3 fatty acids was related to a lower risk of endometriosis. Youseflu et al. [23] demonstrated that the total fat intake was not associated with endometriosis risk. Following the classification of fats, this relationship was found only regarding the consumption of MUFA, docosahexaenoic acid (DHA), and eicosapentaenoic acid (EPA). In contrast, a case-control study [2] found inverse associations between total fat, SFA, MUFA, and TFA consumption and endometriosis risk.

In vitro studies on the survival of endometrial cells in women with and without endometriosis have reported that these cells may be influenced by the fatty acid content of the culture media [27]. Consumption of TFA increases circulating levels of inflammatory markers such as IL-6 and TNF-α, which are involved in endometriosis pathogenesis [28–31]. Activation of inflammatory responses may represent important mediating steps in favoring endometriosis-mediated events [32]. The high content of MUFA makes olive oil less susceptible to oxidation than PUFA [33]. Also in olive oil, most representative phenols are thought to be potent scavengers of superoxide and other reactive species [33], but the positive effects in reducing the risk of endometriosis require further studies. Increased exposure to EPA has been shown to significantly suppress the in-vitro survival of endometrial cells [27], however, cell survival is not affected in cell cultures containing a high proportion of long-chain ω-6 fatty acids (i.e. arachidonic acid) or equal amounts of ω-3 and ω-6 fatty acids [27]. Results of animal studies indicate that ligands of the peroxisome proliferator-activated receptor-γ (PPAR-γ) have been established to induce the reversal of surgically induced endometriosis [34, 35]. TFAs increase the risk of endometriosis through down-regulation of PPAR-γ expression and up-regulation of the effects of cis-PUFAs, which are thought to be a natural ligand for PPAR-γ [36, 37].

We also found no significant association between total fruits and total vegetable consumption and the risk of endometriosis, although the results of the analysis show a declining trend. In a previous report by Youseflu et al. [23], increased consumption of total fruits or total vegetables was associated with a lower risk of endometriosis. Eating more fruits and vegetables reduces circulating levels of inflammatory markers and improves serum antioxidant status [38]. Similarly, increased consumption of vegetables was linked to a reduction in endometriosis risk [7]. Contrary to the results of previous studies, Trabert et al. [2] reported that a high intake of fruits was significantly associated with an increased risk of endometriosis, however, vegetable intake was not associated with endometriosis risk. These findings have been hypothetically attributed to fruit pesticides [39]. In vitro and in vivo studies have displayed that certain class of pesticides may produce estrogenic effects, which promote endometriosis lesions and their recurrence [40–44]. Since inflammation is associated with endometriosis, vitamin C may improve oxidative status by neutralizing free radicals and diminishing endometriosis risk [44].

Analysis of the results of the three studies [2, 21, 23] indicated that there was no significant relationship between legume intake and endometriosis risk. Previous studies have shown that consumption of legumes reduces inflammatory markers such as CRP, TNF-α, IL-6, and other adhesion molecules, as well as the levels of adiponectin [45, 46] that may provide a mechanistic role of legumes in endometriosis.

Similar to other findings, no association was found between the intake of eggs, fish, and poultry and the risk of endometriosis, whereas eating red meat was associated with an increased risk of endometriosis by approximately 17%, with evidence of significant heterogeneity. Likewise, a prospective cohort study reported that women consuming > 2 servings/day of red meat had a 56% higher risk of endometriosis compared to those consuming ≤1 serving/week [12]. According to previous studies, consumption of red meat is directly associated with an increased risk of many chronic diseases such as diabetes, hypertension, fatty liver, cardiovascular disease, and various malignancies [47, 48]. Red meat may be involved in increasing the risk of endometriosis in several ways. One possible mechanism is the effect of red meat on steroid hormones [49]. Epidemiological studies have established that the consumption of red meat is associated with reduced hormone-binding globulin (SHBG) and increased estradiol concentrations [9]. Elevated estrogen levels are involved in inducing inflammatory conditions in endometriosis by stimulating certain prostaglandins [50]. Moreover, animal fats found in meats such as palmitic acid increases endogenous estrogen production and therefore increase endometriosis risk [4, 51]. An additional hypothesis regarding red meat and increased risk of endometriosis relates to the high iron content of meat, as iron is associated with increased oxidative stress and inflammatory status and has also been offered as a possible modulator in endometriosis pathophysiology [52–54].

Energy adjustment is one of the most important modifications in the context of the association between diet and various health issues [55]. In this regard, all of the included studies adjusted dietary energy intake in their statistical analysis to obtain an energy-independent relationship between the consumption of various food groups and the risk of endometriosis, except for the study of Parazzini et al. [7]. However, there was no sensitivity for Parazzini et al. according to the results of influence analysis which implies the integrity of the findings.

### Limitations

There was significant heterogeneity between included studies that may have affected the results and lessened the generalizability of the findings. The probable sources of heterogeneity might be differences in age, BMI, study design, geographical variation, and the quality of the studies. Also, not all studies ensure representative samples of the population and the findings should be interpreted with caution. Patients’ food intake was assessed through an FFQ, which is a subjective estimate of a person’s past intake rather than an assessment of absolute intakes. Furthermore, dietary intakes of participants were reported in different manners, including serving/day or gram/day which could be a source of heterogeneity; however, the lowest values were compared to the highest values to diminish the effects of this issue on the outcomes.

## Conclusion

In conclusion, an optimal intake of total dairy, as well as reduced consumption of red meat, TFA, and SFA may be associated with decreased risk of endometriosis. It may be useful to extend the analysis to other types of food groups and dietary patterns to obtain a complete picture. Additionally, further investigations are needed to clarify the role of diet in the incidence and progression of endometriosis. Cohort studies may be better able to capture long-term intake, especially those that used FFQs administered at multiple time points which need to be done on this topic if future studies.

## Data Availability

The data that support the findings of this study are available from the corresponding author upon reasonable request.
